# Quality of antenatal care in Zambia: a national assessment

**DOI:** 10.1186/1471-2393-12-151

**Published:** 2012-12-13

**Authors:** Nicholas N A Kyei, Collins Chansa, Sabine Gabrysch

**Affiliations:** 1 University of Heidelberg, Institute of Public Health, Im Neuenheimer Feld 324, Heidelberg, 69120, Germany; 2 Department of Policy and Planning, Ministry of Health Headquarters, Ndeke House, Lusaka, 10101, Zambia; 3 Current address: Public Health Division, 37 Military Hospital, Accra, Ghana

**Keywords:** Maternal health services, Prenatal care, Health care quality, Africa South of the Sahara

## Abstract

**Background:**

Antenatal care (ANC) is one of the recommended interventions to reduce maternal and neonatal mortality. Yet in most Sub-Saharan African countries, high rates of ANC coverage coexist with high maternal and neonatal mortality. This disconnect has fueled calls to focus on the quality of ANC services. However, little conceptual or empirical work exists on the measurement of ANC quality at health facilities in low-income countries. We developed a classification tool and assessed the level of ANC service provision at health facilities in Zambia on a national scale and compared this to the quality of ANC received by expectant mothers.

**Methods:**

We analysed two national datasets with detailed antenatal provider and user information, the 2005 Zambia Health Facility Census and the 2007 Zambia Demographic and Health Survey (DHS), to describe the level of ANC service provision at 1,299 antenatal facilities in 2005 and the quality of ANC received by 4,148 mothers between 2002 and 2007.

**Results:**

We found that only 45 antenatal facilities (3%) fulfilled our developed criteria for optimum ANC service, while 47% of facilities provided adequate service, and the remaining 50% offered inadequate service. Although 94% of mothers reported at least one ANC visit with a skilled health worker and 60% attended at least four visits, only 29% of mothers received good quality ANC, and only 8% of mothers received good quality ANC and attended in the first trimester.

**Conclusions:**

DHS data can be used to monitor “effective ANC coverage” which can be far below ANC coverage as estimated by current indicators. This “quality gap” indicates missed opportunities at ANC for delivering effective interventions. Evaluating the level of ANC provision at health facilities is an efficient way to detect where deficiencies are located in the system and could serve as a monitoring tool to evaluate country progress.

## Background

Every day, approximately 800 women die from preventable causes related to pregnancy and childbirth [1]. In 2008 over 300,000 maternal deaths occurred worldwide and almost all of these in low- and middle-income countries [2,3]. It is also estimated that every year 3 million newborn babies die within the first month of life [4] and between 2.1 to 3.8 million babies are stillborn [5-7]. Antenatal care (ANC) is one of the recommended interventions to help reduce these alarming maternal and newborn mortalities [8-10]. Most low-income countries have adopted a focused ANC strategy promoted by WHO which includes four visits and structured elements within these [11-14].

Although the percentage of women attending ANC (for at least one visit) generally tends to be satisfactory even in low-income countries, maternal and neonatal mortality remain high. In Zambia, for example, 94% of women attend ANC at least once, while maternal mortality is estimated at 591 maternal deaths per 100,000 live births, and neonatal mortality at 34 neonatal deaths per 1,000 live births [15]. This weak relationship between ANC use and maternal and newborn survival has motivated a recent call to focus on content and quality of care provided rather than mere ANC attendance as we aim at achieving MDG 4 and MDG 5 [16]. Although researchers frequently highlight the importance of quality of maternal care in improving maternal and newborn health [17-19], the quality of ANC remains insufficiently studied.

Donabedian proposed a framework for assessing quality of care which distinguishes between the attributes of the health care setting (structure), the actual care delivered (process) and the end result of the interaction between an individual and the health care system (outcome) [20-22]. Maxwell added to this initial concept by arguing that quality of care cannot be measured in a single dimension and suggested six dimensions of quality [23]. These quality of care dimensions have been adopted by various organizations [24,25].

Tools to capture several dimensions of quality of ANC received by mothers have been developed for high-income settings [26,27], but are not easily transferable to low-income settings. Studies in low-income countries usually rely on population-based surveys such as the Demographic and Health Survey (DHS) [28], which provide some information on quality of ANC received by mothers, or on community surveys in areas with interventions aiming to improve the quality of maternal health care [29,30] where the quality of care is not necessarily representative for the country or region as a whole.

Little conceptual work has been done on measurement of ANC quality at health facilities [31], although availability of ANC interventions is a prerequisite for women receiving good quality care. In particular, it is necessary that drugs and equipment are available at the facility, that health workers are present and have the necessary knowledge and skills, and that they actually provide the recommended interventions. In settings where health facilities often lack drugs or skilled personnel, evaluating level of service provision may be more diagnostic in terms of identifying where the problem lies, than collecting information from the population on the care received. So far, hardly any published studies have assessed quality of ANC provided at health facilities. One study in Tanzania used health facility information to compare quality of ANC between public and private providers, but was limited to few facilities in an urban area and thus the results are not representative for the country [31].

Possible reasons for this neglect or inadequate measurement of quality of care, besides the conceptual difficulties in capturing the multiple dimensions of quality, include the weakness of routine health information systems, and the subsequent dependence on household surveys, which lack appropriate information on technical aspects of quality of care, as pointed out by Graham and Varghese [17]. Only few low-income countries have detailed information on the services provided by their health facilities and can thus evaluate quality of care at the point of provision.

In Zambia, both a national Health Facility Census from 2005, and a national household survey (Zambia DHS 2007) are available, presenting a unique opportunity to illustrate how the barriers to the measurement of quality of ANC can be overcome methodologically. We draw upon these datasets to comprehensively assess and describe the quality of ANC in Zambia at that point in time, which can serve as a baseline to show any improvements against. The specific objectives were to: (i) assess various quality of care dimensions for ANC facilities in Zambia; (ii) ascertain how many of Zambia’s ANC facilities met developed criteria for optimum and adequate level of service provision, and (iii) assess and compare level of service provision at health facilities with the quality of ANC received by expectant mothers in Zambia.

## Methods

### Data sources

The Health Facility Census (HFC), developed by the Japan International Cooperation Agency, is a national-level assessment of the functionality of health system assets, providing extensive information useful for health system planning [32]. There is no sampling; instead, information is collected on every facility. The 2005 Zambia HFC [33] covered all public and missionary health facilities at all levels in the country, as well as major private facilities, in total over 1400 facilities. The assessment included self-reported availability of interventions, a head count of health workers and verification of the condition of the infrastructure and certain utilities. The Zambian HFC has been used previously to study access to delivery care [34,35].

The 2007 Zambia Demographic and Health Survey (DHS), a nationally representative household cluster survey, interviewed 7,146 women aged 15–49 years and 6,500 men aged 15–59 years across Zambia. All women who had given birth in the five years preceding the survey were asked a range of questions about maternal care. Detailed information about antenatal care was only collected for the last live birth in that period [15]. The dataset contains antenatal information on 4,148 births (counting multiple pregnancies as one birth) that occurred between 2002 and 2007.

### Health facility classification

We adopted a definition of quality based on the frameworks of Donabedian [20-22] and Maxwell [23] and focused on the process attributes of ANC, particularly on the technical aspects of the provider-client interaction, in order to judge whether the new focused ANC requirements [12,36] were fulfilled. Since “structure” is mainly considered as the conduit through which care takes place, rather than a true component of quality of care [37], we only included the availability of skilled health workers into our definition. The availability of certain equipment and drugs are implicit in the performance of certain functions.

Information collected on ANC provision in a health facility included the availability of the service, the number of ANC days per week, performance of screening tests for anaemia, syphilis, urine protein and urine sugar, tetanus toxoid vaccination, folate/iron supplementation, intermittent presumptive treatment (IPT) of malaria, voluntary counseling and testing (VCT) for HIV, prevention of mother-to-child transmission (PMTCT) of HIV, routine discussion of family planning with pregnant women, and ANC outreach services. Based on the recommended interventions for pregnancy care [38], initial data analysis and professional judgment, including feedback from several maternal health researchers and medical practitioners with experience in low-income countries, we developed a framework for assessing the level of ANC provision. This combined availability of five key antenatal functions, five relevant screening tests, availability of skilled health workers according to country definition (doctors, midwives/nurses & clinical officers) [15], as well as availability of other pertinent services in the continuum of care (delivery care, PMTCT), thus representing various dimensions of quality of care as suggested by Maxwell and adopted by WHO [23-25]. In this study, we refer to the quality aspects of ANC provision at health facilities as “level of service provision” to avoid confusion with the quality aspects of ANC received by women as reported in the DHS (next sub-chapter).

We first evaluated the level of service provision for four core ANC quality attributes individually (days per week with ANC provision, availability of skilled health workers, ANC functions, and ANC screening tests) and then combined them to assess the overall level of ANC provision at all Zambian ANC facilities. Table 1 shows the framework used to classify levels of service provision. Although we considered them important, we were unable to include provision of postnatal care, blood group and rhesus testing, provision of ANC outreach services and routine family planning information at ANC due to problems with how the questions were recorded or because these questions were not asked in all facility types in the HFC.

**Table 1:** Framework for the classification of level of ANC provision in Zambian health facilities

**WHO quality of care dimension**	**Process attributes**	**Minimum requirements for optimum level**	**Minimum requirements for adequate level**
**Access to care**	ANC days per week	3+	1
**Responsiveness & Appropriateness**	ANC outreach	No requirement	No requirement
	IPT of malaria	Yes	No requirement
	Folate/iron supplement	Yes	No requirement
	VCT for HIV	Yes	No requirement
	Pre-eclampsia screening	Yes	No requirement
**Continuity of care**	PMTCT service	Yes	No requirement
	Delivery service	Yes	No requirement
	Postnatal service	No requirement	No requirement
	Routine FP information	No requirement	No requirement
**Patient safety**	Skilled health workers registered ^a^	3+	1
	EmOC or EmOC referral capacity	Yes	No requirement
**Effectiveness & Efficiency**	Screening tests: Hemoglobin, Syphilis, Urine protein, Urine sugar, Blood group + Rhesus factor	Any 3+ tests (including urine protein)	Any 1 test
	ANC functions: Folate/iron supplement, Tetanus vaccine, VCT for HIV, PMTCT of HIV, IPT of malaria	All 5 functions	Any 3 functions

^a^ Doctor, nurse/midwife, clinical officer.

VCT, voluntary counseling and testing; IPT, intermittent preventive treatment; PMTCT, prevention of mother to child transmission.

A facility was considered to provide an optimum level of ANC service if providing at least three days of ANC per week, performing at least three screening tests including urine protein testing, offering all five key ANC functions and having three or more skilled health workers registered at the facility, in addition to offering Emergency Obstetric Care (EmOC) referral and delivery services. A facility was classified as providing an adequate level of service if failing in any of the criteria for optimum level but providing at least one day of ANC per week, performing at least one screening test, at least three ANC functions and having at least one registered skilled worker. Failing in any of these criteria, a facility was considered offering an inadequate level of ANC services.

### Quality of received ANC

The 2007 Zambia DHS collected details on ANC received in the five years prior to the survey for all last births to the mothers interviewed [15]. Information was available on 4148 births and was used to describe the characteristics of ANC received by expectant mothers in Zambia. This included information on ANC use, type of ANC provider, place of ANC provision, number of ANC visits, timing of first ANC visit and on the interventions received. Information was available on the following interventions [15]: weight measurement, height measurement, blood pressure measurement, urine sample taken for analysis, blood sample taken for analysis, offered VCT, iron supplementation provided, antimalarial drug provided for IPT, birth preparedness plan discussed, treatment provided for intestinal parasites and tetanus toxoid vaccination. In the few instances where information was missing on an item or mothers did not know, we assumed a skilled provider was not seen, the intervention was not received, etc.

Having received “good quality ANC” was defined as having attended at least the recommended four ANC visits with a skilled provider and received at least eight antenatal interventions, while our definition of “moderate quality ANC” required four visits with a skilled provider and five to seven antenatal interventions.

## Results

### Level of ANC service provision at Zambian health facilities

Out of the 1391 health facilities with information on the availability of ANC in the Health Facility Census (HFC) dataset, 1299 (93%) were recorded as providing ANC. Of these, 71 (5.5%) were health posts, 950 (73%) rural health centers, 207 (16%) urban health centers, 52 (4%) first-level hospitals, 18 (1.4%) second-and-third-level hospitals, and one ANC facility was unclassified. The majority of ANC facilities were run by the Zambian government (85%) and by churches or NGOs (9%).

Of the 1299 ANC facilities in Zambia in 2005, 42% provided ANC once a week, 30% twice a week and 27% three days or more per week. For 12 ANC facilities “zero” days of ANC per week were recorded. Five of the latter provided ANC outreach services; another reason for this seemingly contradictory statement could be that ANC is provided less frequent than weekly in these facilities.

Table 2A shows the availability of relevant ANC functions, screening tests and related services in Zambian ANC facilities. While folate/ iron supplementation, tetanus vaccination and IPT of malaria were provided by the vast majority of ANC facilities, detection and prevention of mother-to-child transmission of HIV was only available at a third of ANC facilities at the time of the census. Most screening tests were not commonly available: only 16% of ANC facilities provided hemoglobin testing which is helpful in diagnosing anemia, and only half provided syphilis testing. Urine protein testing, which is important for detecting hypertensive complications of pregnancy such as pre-eclampsia, was performed by less than a quarter of ANC facilities. The vast majority of ANC facilities also provided family planning, delivery and postnatal care, thus ensuring continuity of care.

**Table 2:** ANC interventions provided by facilities and received by mothers in Zambia

**A) Interventions provided**	**Facilities (%) n=1299**	**B) Interventions received**	**Mothers (%) n=4052**^**c**^
Folate/iron supplementation	99.1	Folate/iron supplementation given or bought	93.2
Tetanus vaccination	86.4	Tetanus vaccination received	81.2
VCT for HIV	33.0	VCT for HIV offered^d^	54.1
PMTCT against HIV spread	30.5		
IPT of malaria	97.2	IPT of malaria taken	88.8
		Drug for intestinal parasites taken^e^	36.7
		Weighed	91.4
		Height measured	26.5
		Blood pressure taken	82.5
**Screening tests**			
Hemoglobin	15.8	Blood sample given^f^	60.8
Syphilis	49.8		
Urine protein	22.6	Urine sample given	22.6
Urine sugar	20.8		
**Other related services**			
ANC outreach	81.6		
Postnatal service^a^	94.8		
Delivery service	84.8		
EmOC or EmOC referral^b^	98.7		
		Birth preparedness plan discussed	73.6

EmOC, Emergency Obstetric Care; IPT, Intermittent presumptive treatment; PMTCT, Prevention of mother to child transmission; VCT, Voluntary counseling and testing; ^a^n=1275, ^b^n=1070 (not asked if no delivery care), ^c^women attending ANC; less than 10 missing/unknown values for all items (counted as not received), except ^d^44, ^e^19, ^f^12 missing or unknown.

Table 3 shows the distribution of core service provision attributes (ANC days per week, skilled health workers, ANC functions and ANC tests) separate for the different facility types. Not surprisingly, health posts and rural health centers provided the least number of ANC interventions, urban health centres and first-level hospitals were in between, and second-and-third-level hospitals provided most.

**Table 3:** Core service provision attributes at Zambian ANC facilities, by facility type

	**Health posts**	**Rural health centers**	**Urban health centers**	**Level 1 hospitals**	**Level 2&3 hospitals**	**Total**^**a**^
**Total No**	71	950	207	52	18	1299
**ANC days per week**						
0	1%	<1%	<1%	8%	6%	1%
1-2	80%	79%	44%	48%	50%	72%
3+	18%	20%	55%	44%	44%	27%
**Registered SHWs**						
None	11%	14%	1%	2%	0	11%
1-2	83%	62%	10%	0	0	51%
3+	6%	23%	90%	96%	100%	37%
Missing info	0	1%	0	2%	0	1%
**ANC functions provided**						
<3	24%	13%	3%	15%	6%	12%
3-4	69%	76%	44%	33%	28%	68%
All 5	7%	11%	53%	52%	67%	20%
**ANC tests provided**						
None	65%	53%	12%	10%	6%	45%
1-2	32%	38%	48%	21%	6%	39%
3+	3%	9%	40%	69%	89%	17%

(Percentages may not exactly add up to 100% due to rounding).

^a^ Facility type missing for one ANC facility.

Applying our classification framework for assessing levels of service provision (Table 1), only 45 of 1299 Zambian ANC facilities (3%) fulfilled the criteria for optimum level of ANC provision, while 608 ANC facilities (47%) were classified as providing an adequate level of service, leaving 646 facilities (50%) in the category of inadequate level of service provision.

**Figure 1:**
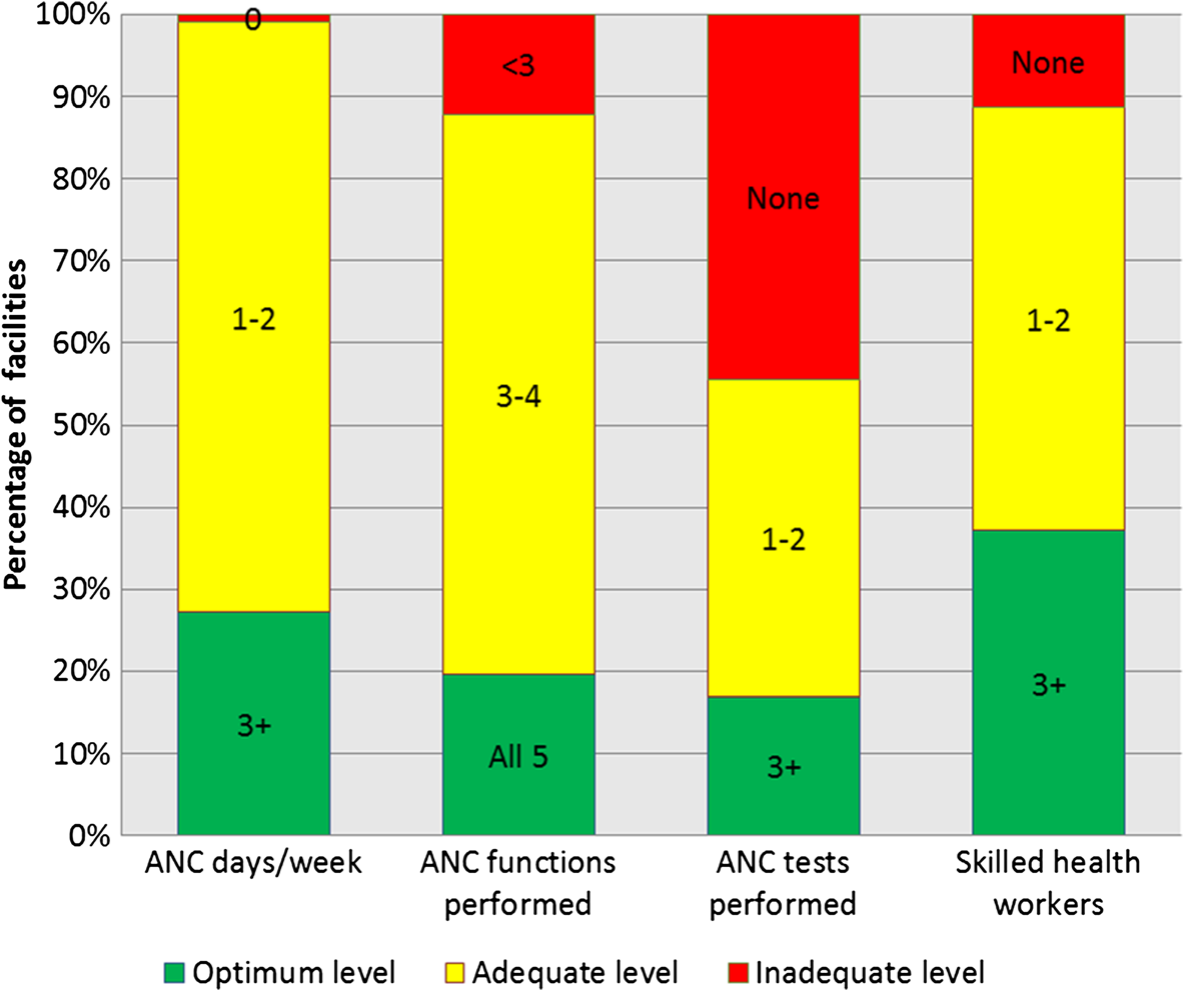
**Core ANC service provision attributes at Zambian ANC facilities (n=1299).** The percentage of Zambian ANC facilities fulfilling four core antenatal service provision attributes is shown separately. Most facilities offer ANC 1–2 days per week and provide 3–4 ANC functions, both criteria for adequate level of service provision. However, nearly half of all ANC facilities do not offer any ANC screening tests which was considered inadequate. For each attribute, less than 40% of facilities fulfilled the criteria for optimum level of ANC provision.

**Figure 2:**
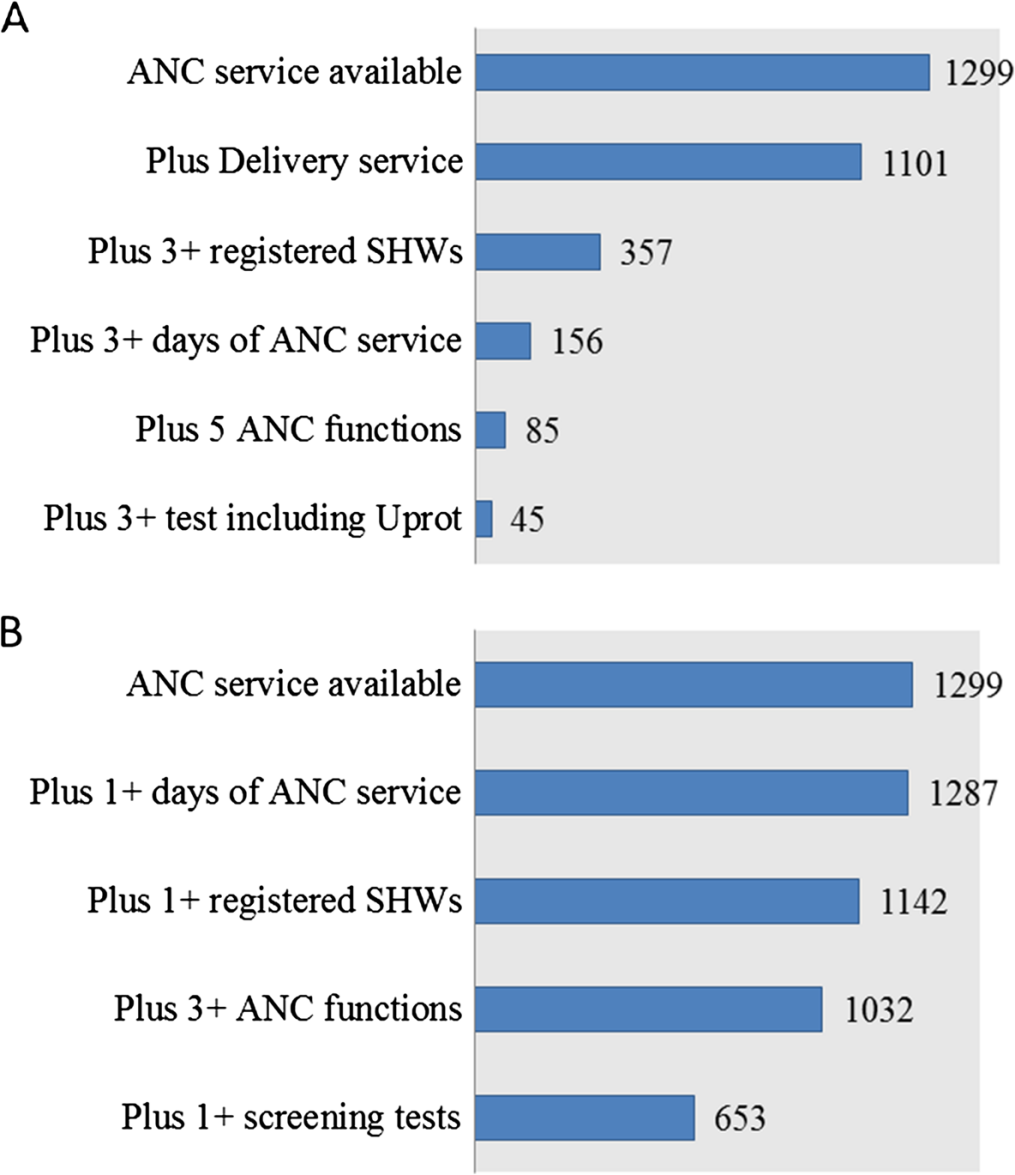
**Cumulative presence of minimum requirements for levels of ANC provision at Zambian health facilities.** This figure illustrates the effect of combining various attributes required for optimum level (A) or adequate level (**B**) of service provision. Of 1299 ANC facilities, 1101 also offered delivery services, considered important for the continuum of care. Out of these, 357 employed at least 3 skilled health workers (SHW) and 156 also had services 3 or more days a week. Only 45 also offered 5 ANC functions and at least 3 screening tests and were thus categorised as optimum level of ANC provision (**A**). Nearly all ANC facilities opened once a week and 1142 also had 1 skilled health worker. Of these, 1032 offered at least 3 ANC functions and 653 also offered at least one screening test, thus fulfilling all minimum requirements for adequate level of ANC provision (**B**). Uprot= Urine protein test.

**Figure 3:**
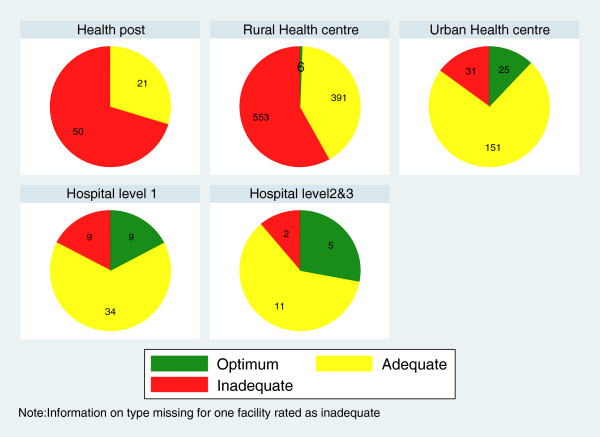
**Level of service provision at Zambian ANC facilities (n=1299), by facility type.** Hospitals offered mainly optimum or adequate ANC, as did urban health centres. More than half of rural health centres, the main ANC providers, and of health posts provided an inadequate level of service.

Figure 1 shows the distribution of the core antenatal service provision attributes separately, while Figure 2 illustrate the effect of combining various attributes of optimum or adequate care. Figure 3 shows that level of provision varies greatly between facility types with the majority of health posts and rural health centers providing inadequate ANC service.

### Quality of ANC received by Zambian women

While 98% of the 4148 women in the Zambia DHS 2007 attended ANC at least once and 94% attended at least once and saw a skilled health worker, only 60% had the recommended four antenatal visits and 58% received the recommended ANC of at least four visits with a skilled health worker. Only 19% of mothers who attended ANC had their first ANC visit in the first trimester, while 74% attended for the first time during the second trimester. Most mothers (72%) attended ANC at a health center and the vast majority (91%) received care from a nurse or midwife (Table 4).

**Table 4:** ANC use, timing of first ANC visit, ANC place and provider in Zambia

	**Percentage**
**Number of ANC visits (n=4144)**	
None	2.3
1	2.2
2	8.0
3	25.9
4	28.9
5+	31.7
Don’t know	1.1
**Month of first ANC visit (n=4050)**	
1-2	3.5
3	15.1
4	27.1
5	28.4
6	18.0
7	6.0
8-9	1.5
Don’t know	0.3
**ANC place (n=4048)**	
Government hospital	11.3
Private hospital/clinic	1.1
Mission hospital/clinic	5.0
Health center	71.9
Health post	8.2
Own or other home	0.5
Other	2.0
**Highest ANC provider (n=4052)**	
Doctor	1.8
Nurse/midwife	90.5
Clinical officer	3.4
TBA	2.9
Other	1.4

When looking at content of care as reported in the 2007 Zambia DHS, certain ANC interventions were commonly received by Zambian mothers while others were not (Table 2B). Iron supplementation, weight measurement, IPT of malaria, blood pressure measurement, and tetanus vaccination were each received by over 80% of women, while VCT for HIV was received by half, drugs for intestinal parasites by about a third, and only about a quarter of women reported that their urine had been tested at ANC. Approximately half of the mothers received eight or more ANC interventions, 40% received five to seven interventions and 12% received less than five interventions.

**Figure 4:**
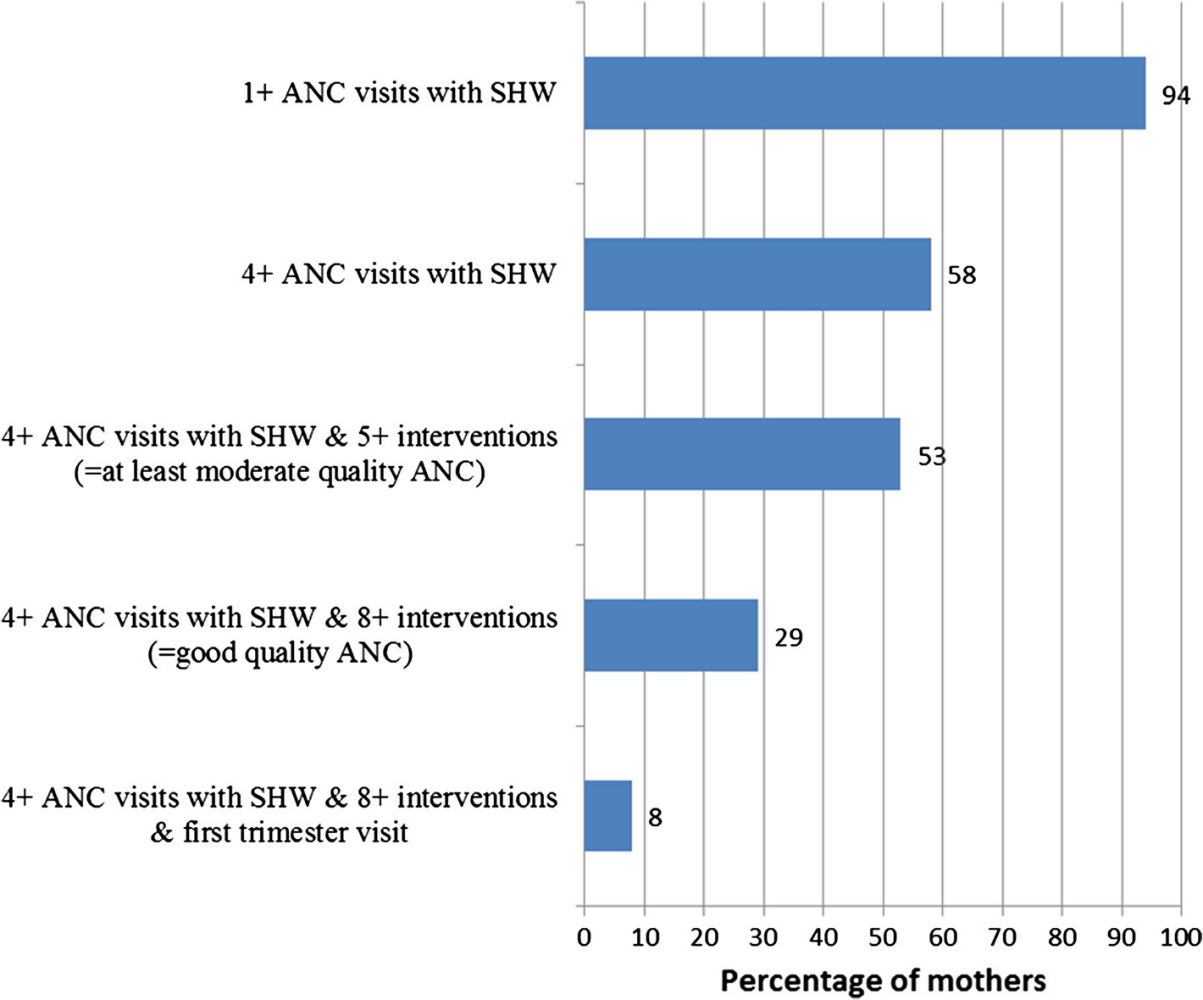
**Quality of ANC received by Zambian mothers (n=4148).** Although 94% of mothers had at least one ANC visit with a skilled health worker and 58% had at least four visits – the indicators commonly tracked -, only 29% of mothers in Zambia received good quality ANC and only 8% received good quality ANC and attended in the first trimester.

Overall, 29% of mothers were judged to have received good quality ANC, defined as at least four ANC visits with a skilled provider and having received at least eight ANC interventions, and 24% of mothers received moderate quality ANC, defined as at least four visits with a skilled provider and having received five to seven ANC interventions (Figure 4), resulting in 53% of mothers receiving good or moderate ANC. Only 8% of mothers received good ANC and attended ANC in the first trimester.

## Discussion

Drawing upon two national datasets with detailed provider and user information, the Zambia 2005 HFC and the Zambia 2007 DHS, we demonstrated that although ANC attendance in Zambia was high, insufficient provision of important antenatal screening tests and interventions were limiting the level of ANC delivered, which was reflected in the high proportion of women not receiving good quality ANC. To our knowledge, this is the first study to present data on ANC quality at health facilities on a national scale and the first to develop a tool for classifying the level of ANC service provision in health facilities in low-income settings that includes several quality dimensions.

Rural health centers, the main ANC providers, usually opened at least once a week for ANC, employed at least one skilled health worker and provided at least three ANC functions, however, more than half did not provide any ANC screening test. In contrary, almost all the hospitals had at least three skilled health workers and the majority of them performed all five essential ANC functions as well as three or more screening tests. Hospitals, however, form the minority of ANC providers in Zambia and are used by less than 20% of expectant mothers.

Various important ANC interventions that could contribute to improving maternal and newborn health remain largely unavailable in Zambia. Only 23% of ANC facilities were able to perform a urine protein test, reflected by a similarly low number of mothers reporting to have had their urine checked. Coupled with the fact that 17% of mothers did not have their blood pressure measured, this will make it difficult to detect and manage hypertensive complications of pregnancy before potentially fatal eclamptic fits develop. This defeats the objective of focused ANC, which hinges on early detection and treatment of complications [14].

Besides early detection and treatment of pre-eclampsia, screening for syphilis with treatment of positive pregnant women and screening for HIV infection in order to implement PMTCT are effective interventions that are recommended for low-resource settings [11]. Despite the high HIV prevalence of 16% among women of reproductive age in Zambia [15], only 30% of ANC facilities offered VCT and PMTCT to pregnant women and only half of mothers reported to have been offered this service. It is evident that the new model integrating VCT and PMTCT into ANC had not been fully implemented by the time of the 2005 Zambia HFC. Syphilis prevalence among women aged 15–49 in Zambia was 4% at the time of the DHS survey [15], and it is estimated that 69% of pregnant infected women will have an adverse pregnancy outcome [39]. Improving the coverage of antenatal syphilis screening could reduce the syphilis-attributable incidence of stillbirth and perinatal death by 50% [39]. However, implementation was still a big challenge in Zambia in 2005 as our data showed that only about half of the country’s ANC facilities offered syphilis screening then.

Using information both from ANC providers and ANC users enabled us to get a complementary picture of ANC quality in Zambia. Unfortunately, we did not have information on facility case load in the HFC. Knowing how many women sought ANC in each facility could contribute towards understanding the reasons why most women in Zambia did not receive good quality ANC. For instance, one could establish whether women in an area used certain inadequate facilities at all, and in case women mostly used facilities offering an optimum level of service but still did not receive such care, this would indicate that facilities were not always providing available ANC functions or screening tests. This information would help to differentiate between the problems of availability of services and correct provision of available services, which require different approaches to their solution.

In interpreting our findings, it is worth noting certain limitations of this study. The Zambian HFC, on which our assessment of levels of service provision is based, only provides a snapshot of the health facilities at the time of visit in 2005. The situation now is likely to be different and possibly improved. With assistance from the World Bank, the Japan International Cooperation Agency and the UK Department for International Development, the Zambian Ministry of Health has bought necessary equipment in recent years which will have improved structural quality. A number of new health facilities have been constructed and a direct entry mid-wifery programme has made it possible to produce, recruit, and place midwives at a rapid rate. A results-based financing project targeting maternal and child health is also contributing to improve the situation [40-44].

Additionally, important information such as opening hours, training of health workers, and client satisfaction were not collected in the HFC and could thus not be included in our quality classification. Proxy indicators such as ANC days per week and the availability of skilled health workers were included in assessing level of provision instead. The assumption that provision of certain interventions implies availability of the necessary equipment and drugs also does not necessarily hold; providers may improvise and provide an intervention in a suboptimal way. There was furthermore no information on whether interventions were given in the correct way or information on provider-client interaction, as the HFC - given its national scope, covering around 1400 facilities - did not allow observation of service provision. For these reasons, it is likely that some facilities have been wrongly classified in terms of level of ANC provision. We unfortunately also did not have any complementary qualitative information, e.g. from key informant interviews which would have helped to interpret the quantitative findings. This is lamentable because perceived quality of care by mothers as well as other qualitative factors have been shown to be critical determinants of ANC uptake [45]. Finally, mothers may not have correctly remembered the details of the ANC they received, causing measurement error in the user information; this was reduced by restriction to the most recent birth in the DHS.

## Conclusions

In summary, very few facilities in Zambia provide an optimum level of ANC services, as judged by the criteria we developed using technical process of care attributes, and this is reflected in the less than optimal quality of ANC received by the majority of expectant mothers.

Although 94% of mothers had at least one ANC visit with a skilled health worker and 60% had at least four visits – the indicators tracked by “Countdown to 2015” [16] -, only 29% of mothers in Zambia received good quality ANC and only 8% received good quality ANC and attended in the first trimester (Figure 4). “Effective coverage”, defined as “the proportion of the population who needed a service that received it with sufficient quality to be effective” [46,47], is thus much lower than suggested by current service coverage indicators. This “quality gap” [48,49] is an indication that there are still many “missed opportunities” at ANC for delivering effective interventions to improve maternal and newborn health [13,50].

There have been numerous calls to “improve the content and quality of services offered” at ANC [13,17,19,49,50] and to develop indicators that capture quality of care [17-19,46,51]. We have shown that it is possible to develop an indicator of “effective ANC coverage”, using information on interventions received from the DHS. Furthermore, we have demonstrated that evaluating level of ANC provision in health facilities can be enlightening and point towards deficiencies that should be addressed. It has been shown that to improve the quality of ANC received by mothers, efforts should focus on both improving the level of services provided at ANC facilities and their accessibility [52]. Especially in settings where services are highly deficient, collecting data upstream at health facilities is advisable. Health Facility Censuses could be adapted for continuous monitoring and could then provide actionable data at the local level at shorter intervals and probably lower cost than population surveys.

## Ethics

This study was granted ethical approval by the London School of Hygiene & Tropical Medicine ethics committee (application number 5172).

## Abbreviations

ANC: Antenatal care; DHS: Demographic and Health Survey; EmOC: Emergency Obstetric Care; HFC: Health Facility Census; HIV: Human Immunodeficiency Virus; IPT: Intermittent Presumptive Treatment; MDG: Millennium Development Goal; PMTCT: Prevention of Mother-To-Child Transmission; VCT: Voluntary Counselling and Test; WHO: World Health Organization.

## Competing interests

The authors declare that they have no competing interests.

## Authors’ contributions

NK analysed the data and drafted the manuscript. SG identified and cleaned the data, supervised data analysis and contributed to writing the manuscript. CC provided country-specific input and revised the manuscript. All authors read and approved the final manuscript.

## Pre-publication history

The pre-publication history for this paper can be accessed here:

http://www.biomedcentral.com/1471-2393/12/151/prepub
